# Comparative Analysis of Energy Viability of Crop Residue from Different Corn Varieties

**DOI:** 10.3390/ma18071683

**Published:** 2025-04-07

**Authors:** Raquel García-Mateos, María Teresa Miranda, José Ignacio Arranz, Pilar Romero, Francisco José Sepúlveda, Santiago Cuellar-Borrego

**Affiliations:** 1 Scientific and Technological Research Centre of Extremadura (CICYTEX), C/Pamplona 64, 06800 Mérida, Spain; raquel.garcia@juntaex.es (R.G.-M.); santiago.cuellar@juntaex.es (S.C.-B.); 2 Department of Mechanical, Energy and Materials Engineering, Industrial Engineering School, University of Extremadura, Av. Elvas s/n, 06006 Badajoz, Spain; jiarranz@unex.es (J.I.A.); pmunoz@unex.es (P.R.); fsepulveda@unex.es (F.J.S.)

**Keywords:** corn crop, thermogravimetry, calorimetry, emissions, biofuel

## Abstract

The valorization of agricultural residues assumes a pivotal position in the circular economy by transforming waste into a useful and environmentally friendly product, with the cultivation of corn, as one of the world’s predominant crops, being crucial. This article aimed to investigate the feasibility of using residues from corn crop as biofuels, going more in-depth into determining the effect that crop variety may have on its thermal qualities. Specifically, 12 samples of corn crop residues were studied in three main groups: conventional, forage, and transgenic varieties. To achieve this, proximate and ultimate analyses, thermogravimetric analyses, and differential scanning calorimetry were conducted, along with a study of gas emissions and a statistical comparison of different varieties. From the results obtained, it is worth highlighting the low ash content in all the samples (between 5.55% and 8.42%) and high calorific values (higher than 17 MJ/kg in all cases), as well as optimal thermal results for all the samples studied in both pyrolysis and combustion processes. Significant differences were found between the different varieties; in particular, it was observed that the forage variety presented more optimal conditions for its application in both processes. This may represent a potential competitive advantage for the forage varieties.

## 1. Introduction

The concept of circular economy has gained pivotal significance on both a global scale and within Europe in recent times. In a world where natural resources are limited and concerns about climate change are escalating, the circular economy emerges as a sustainable alternative to the conventional linear economic model. Its main objective is to reduce the extraction of natural resources and minimize the generation of waste, emphasizing practices such as reuse, recycling and remanufacturing [1,2,3,4,5]. This approach not only contributes to the conservation of valuable resources but also mitigates carbon emissions and minimizes pollution.

At the core of the circular economy is the acknowledgment that natural resources are limited. The overexploitation of these resources, exemplified by the case of fossil fuels, has resulted in issues such as scarcity, environmental degradation, and the depletion of crucial ecosystems. In this context, the circular economy emerges as a rational and sustainable solution aimed at preserving and utilizing these resources more efficiently [6,7]. Waste valorization is an essential component of circular economy [1,8]. Rather than viewing waste as something to be disposed of, it is perceived as a potential resource. Specifically, the valorization of agricultural residues has gained significance in the pursuit of more sustainable agricultural practices. Agricultural waste, including crop residues and straw, constitutes a valuable reservoir of organic materials with the potential for reuse. Agricultural biomass can be transformed into bioenergy, helping to reduce dependence on fossil fuels, reducing environmental impacts, and contributing to the rural economy.

At the European level, the utilization of agricultural waste for use as bioenergy is included in the waste hierarchy established in Directive (EU) 2023/2413 of the European Parliament and of the Council of 18 October 2023 [9] as a measure to ensure that energy from biomass is produced in a way that minimizes undue distortionary effects on the biomass feedstock market and adverse impact on biodiversity, the environment, and the climate. In the case of agricultural residues, reuse and recycling prevail over bioenergy according to the hierarchy set out in the Directive, but both options are currently not viable for corn residues. In the case of corn crop residues, as it is the conversion of a residue into biomass, it is not affected by indirect land use change (ILUC) [9], as the main purpose of the crop is its food use, not its use as biofuel, and therefore there is no increase in carbon emissions. In addition, research amply shows that the use of biofuels reduces greenhouse gas emissions. Nordin et al. [10] studied the reduction of emissions through the use of biofuel, taking into account that this reduction will be strongly influenced by the emissions derived from the transport of such biofuel. Kamran et al. [11] focused their research on a food processing plant, in which a biomass consumption system was studied, quantifying a potential reduction in CO_2_ emissions of 2784 tons. Yu et al. [12] determined a value for the agricultural GHG emission reduction potential of bioenergy from marginal lands of almost 50% of total emissions during the study period.

The cultivation of corn plays a critical role in both global food systems and the economy. As one of the most extensively cultivated crops worldwide, corn serves as a crucial food source for both humans and animals. Maize provides essential calories, protein, and nutrients and is used in a variety of products, from staple foods to biofuels. Beyond its significance in sustenance, the cultivation of corn functions as an economic catalyst for farmers and rural communities. It not only generates employment opportunities but also contributes substantially to economic stability.

Globally, the significance of corn cultivation is clearly discernible through an examination of data provided by the Food and Agriculture Organization (FAO) [13]. This crop ranks second in global in terms of both tons of production and hectares of cultivated area. Analyzing the data series spanning from 2011 to 2021 reveals an upward trend in both aspects. The production quantity surged from 887 million tons in 2011 to 1163 million tons in 2022. Similarly, the cultivated area expanded from 172 million hectares in 2011 to 203 million hectares in 2022.

Based on the available data, it is evident that the volume of waste produced by the crop is substantial, emphasizing the critical importance of valorizing this waste. Corn cultivation generates a considerable amount of agricultural residues, including stalks, leaves, and crop residues, and these materials can be effectively utilized as biofuels, thereby mitigating the potential adverse effects associated with the crop. Quantification of residues generated by corn cultivation is challenging due to the influence of numerous factors such as crop rotation, seasonal timing, tillage practices, soil properties, climate, geographical location, and slope. Some studies suggest that approximately 50% of plant biomass consists of residues, although the specific parameters used to determine this figure remain unspecified [14,15,16,17]. Currently, these wastes are left on the soil, being mechanically ground in order to be mixed with the soil after harvest, or used to produce bales for its use in biomass power plants [18].

When considering the varieties of corn crops, there are primarily two distinct categories: conventional corn, intended for human consumption, and forage corn, utilized as animal feed. Conventional corn is cultivated for diverse purposes, serving human consumption and meeting demands in food industry. In the case of forage corn variety, although it is specifically grown to address the nutritional requirements of livestock, utilized either as fresh fodder or as part of silage, it also finds application as a feedstock for industry. The global ratio between cultivated areas and production quantities of conventional corn versus forage corn exhibits considerable variation and is influenced by several factors, including market demands, regional agricultural practices, and government policies. Notably, regions where corn constitutes a pivotal food source tend to allocate a considerable proportion of corn for direct human consumption. In addition, the existence of transgenic corn introduces a new category in which corn is genetically engineered to confer specific desirable characteristics, such as pest resistance, herbicide tolerance, or improved nutritional content. Although globally there has been an increase in the percentage of areas cultivated with this type of corn, this increase has not been as significant in Europe due to stringent health control standards and regulations [19,20].

Studies on the valorization of agricultural residues are essential in the search for more sustainable agricultural practices. The study of different agricultural residues for use as biofuels has been extensively researched previously [21,22,23,24,25,26,27,28]. The results obtained lead to the conclusion that the use of these residues as biofuel is a suitable valorization process. Recently, studies have gone further and tried to determine whether the variety of the particular crop can influence the characteristics of the residue as biofuel. Paniagua et al. [8] studied the cultivation of poplar for use as biofuel, and specifically focused on pyrolysis processes, Mishra et al. [29] focused on the study of different varieties of paddy straw biomass, Stolarski et al. [30] investigated the different biomasses from willow cultivation, whereas Colauzzi et al. [31] concentrated on the study of sorghum varieties for biomass. The study of corn varieties with the aim of using their residues to produce biofuels is essential in the search for sustainable energy sources. Oslaj et al. [32] conducted a study of three corn hybrids for the optimization of biogas production. However, there are no studies in which an exhaustive comparative analysis of the waste of the different corn varieties currently on the market is addressed, taking into account the final use of the same, whether for human or animal consumption, nor if there are differences between them when it comes to the valorization of their waste as biofuel, which is a novelty with respect to previous research. In addition, the varieties were tested under the same weather conditions and on the same soil, which allows these influencing factors to be eliminated from the study. Comparing the thermal properties of the residues from different varieties of corn can provide valuable information when determining whether any of them may have some kind of competitive advantage over the others, as there may be possible options for densification of the residue or better characteristics as biofuel.

When studying the viability of waste for use as biofuel, it is essential to know how it behaves in pyrolysis processes with the absence of oxygen and in conventional combustion processes [33]. This is why this research has been conducted both in an inert atmosphere and with synthetic air, with a view to subsequently defining the most optimal process depending on the type of corn waste encountered.

In addition, for this study, the thermogravimetric study, differential scanning calorimetry, and emission studies by mass spectrometry were carried out, considering that all these techniques are essential and widely used when studying the characteristics of a solid biofuel.

The thermogravimetric study (TG) is used to investigate the thermal properties of a sample during its exposure to controlled temperature variations. This method is based on the precise measurement of the mass variation of a sample as a function of temperature or time. Differential scanning calorimetry (DSC) is a technique employed to determine the variation of heat flows emitted or absorbed by a sample when exposed to a temperature programmed in a controlled atmosphere. The process involves heating a sample and a reference at the same rate and measuring the heat difference between them. DSC is valuable for characterizing thermal changes in a sample. Mass spectrometry (MS) is an analytical methodology employed for the identification of chemical compounds in a sample by assessing the mass of their ions. When applied to biomass samples, it facilitates an examination of emissions to determine potential harmful compounds released in a process. The combination of the three aforementioned techniques provides a comprehensive characterization of the residue under study enabling the delineation of its most optimal utilization as a biofuel.

This work aims to optimize the valorization of the residue generated in the different cultivation corn varieties, categorized by the type of corn they produce, whether conventional, forage, or transgenic. The waste under study was the corn stover produced after the harvest, that is, the corncob and the stalk of the plant. In addition, it will be evaluated whether the corn type is a determining factor for the energetic behavior of the residue and, therefore, should be considered in its application as biofuel. Identifying a variety with significantly better characteristics can improve the economic performance of that variety and increase the potential interest of growers.

## 2. Materials and Methods

### 2.1. Sample Collection and Preparation

For this research, 12 different corn crop samples were carefully selected. These samples were cultivated in 2021 in the Scientific and Technological Research Centre of Extremadura (CICYTEX), within the municipality of Guadajira, located in Badajoz, Spain.

Annually, as part of the agronomic evaluation of the new conventional and transgenic varieties of corn for grain, carried out by the Spanish Ministry of Agriculture, CICYTEX carries out a corn crop evaluation to assess the adaptation of the new corn varieties in Spain, particularly in each of the producing areas. To this end, a plot was selected for corn cultivation where different varieties were sown, with the aim of determining the production results as well as other agronomic parameters of the different varieties, all under the same growing conditions, in order to carry out a full-scale comparison. Among the varieties sown, specific varieties were selected for study as well as others known to be used as planting controls. The most important parameters studied in the agronomic evaluation are yield, plant density, plant height, height of the cob insertion node, and stay-green among others. As an additional study to that evaluation, the present research served to determine the viability of the residues of the different corn varieties as biofuel, and the influence of the type of corn on the thermal characteristics of the residues.

These corn varieties can be categorized into three distinct groups, from those mentioned above, based on their intended use. The first group comprises conventional corn varieties intended for human consumption, encompassing five distinct varieties designated as C1, C2, C3, C4, and C5. The second group consists of four forage corn varieties designed for animal consumption, identified as F1, F2, F3, and F4. The third and final group consists of three transgenic corn varieties, which have been labelled as T1, T2, and T3.

In alignment with the research’s objectives, complete corn plants were harvested, and a comprehensive analysis of the entire plant was conducted. The corn cobs were carefully shelled, and the plant was harvested directly without contact with the soil, to ensure that the stover remained unaltered and unaffected by any potential degradation from prolonged soil exposure.

Samples of corn plants were collected randomly from each cultivation area of the different corn varieties throughout the crop area and stored in sacks for transport to the laboratory. Once at the laboratory, the corn kernels were separated from the rest of the plant and the residual plant was crushed. The crushed product was then stored in insulated plastic bags so that the moisture content of the samples would not be affected. The proximate and ultimate analyses were conducted immediately after crushing to minimize any alterations that might occur in the residue and thus obtain the true characteristics of the residue.

To prepare the samples for analysis, a hammer mill (CIP Line SG40, Vezza d’Alba, Italy) equipped with a 5 mm sieve at the outlet was used. In the case of samples used in TG/DSC analyses, a ball mill (Retsch MM 301, Haan, Germany) was subsequently used until the samples were practically pulverized.

**Figure 1:**
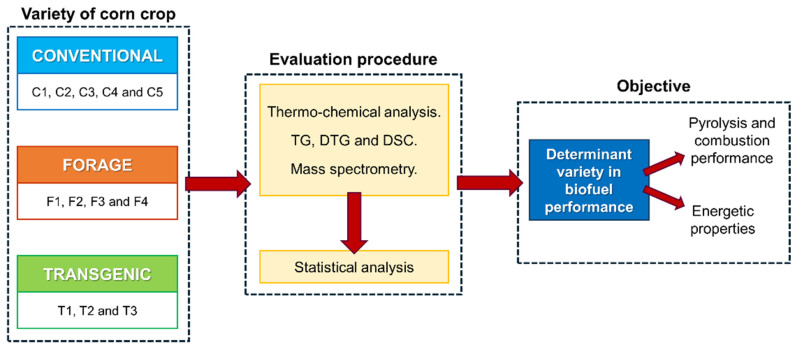
Methodological diagram.

Figure 1 shows the methodology used in this study.

### 2.2. Thermo-Chemical Analysis

To assess the fuel properties of residues from the 12 corn samples a comprehensive analysis was conducted. This involved determining proximate and ultimate analyses, as well as the calorific value.

The moisture content was calculated following the guidelines outlined in standard ISO 18134-2:2017 [34].

For both, ultimate and proximate analyses standards ISO 16948:2015 [35] and ISO 16994:2016 [36] were applied. In the case of volatile matter content, percentage was determined according to the standard ISO 18123:2016 [37], whereas ash content was determined following the procedures laid out in standard ISO 18122:2015 [38]. The percentage of fixed carbon was derived by subtracting 100% from the sum of volatile matter and ash on a dry basis [39,40]. For this, a muffle furnace Hobersal CRN-58 PAD P was used. All samples were run in triplicate to ensure adequate reproducibility of the results, and the results reported are means with standard deviations.

In the determination of the higher heating value, a bomb calorimeter IKA C2000 Basic was employed in compliance with standard ISO 18125:2017 [41]. All samples were run in triplicate to ensure adequate reproducibility of the results, and the results reported are means with standard deviations.

### 2.3. Thermogravimetric and Differential Scanning Calorimetry Analyses

A NETZSCH thermobalance model STA 449F5 Jupiter (NETZSCH, Selb, Germany) was used for the thermogravimetric (TG) and differential scanning calorimetry (DSC) analyses. Aluminum crucibles (Al_2_O_3_) were used for the tests and a heating rate of 20 °C/min from 40 °C to 900 °C was selected. For the pyrolysis study, the gas used was argon with a flow rate of 50 mL/min, and for the combustion study, air atmosphere (80/20) was used with a gas flow rate of 50 mL/min. In both cases, argon was used as a protective gas with a flow rate of 20 mL/min. All samples were run in triplicate to ensure adequate reproducibility of the results and results reported included standard deviations. Prior to the tests, the thermobalance was calibrated using the calibration kits provided by NETZSCH, specifically using indium (In), tin (Sn), bismuth (Bi), zinc (Zn), aluminum (Al), and gold (Au), with known melting points.

For the determination of the different parametric ranges of the experimentation, the results extracted in different previous research found in the different bibliography were studied [42,43,44,45], as well as in our previous experience of thermogravimetry of other agroforestry residues [18,46,47,48]. In all the references reviewed, it was observed that the biomass conversion processes, either in pyrolysis or combustion processes, ended below 600 °C. Therefore, the maximum temperature range of 900 °C ensures that such conversion occurs.

On the basis of TG, the calculation of the differential thermogravimetric curve (DTG), corresponding to the first derivative of the TG curve, has been carried out. To analyze the mass loss process and reaction rates during pyrolysis and combustion, as well as to determine the different temperature events during both processes, the tangent method widely used in different scientific works have been considered [49,50,51,52,53].

For the study of the pyrolysis process, the pyrolysis index, P_i_, end pyrolysis index, P_f_, and pyrolysis characteristic index, P were calculated [8].

(1) $$P_{i}=\frac{{DTG}_{max}}{t_{p}t_{m}}$$

(2) $$P_{f}=\frac{{DTG}_{max}}{\Delta t_{1/2}t_{p}t_{f}}$$

(3) $$P=\frac{{DTG}_{max}{DTG}_{mean}}{T_{m}^{2}T_{f}}$$

The meaning of each term in the formulas are shown in Table 1:

In the case of combustion process, the ignition index, D_i_, burnout index, D_f_, devolatilization index, D, and combustion characteristic index, S, were calculated [49,54].

(4) $$D_{i}=\frac{{DTG}_{max}}{t_{p}t_{i}}$$

(5) $$D_{f}=\frac{{DTG}_{max}}{\Delta t_{1/2}t_{p}t_{f}}$$

(6) $$D=\frac{{DTG}_{max}}{T_{p}\Delta T}$$

(7) $$S=\frac{{DTG}_{max}{DTG}_{mean}}{T_{i}^{2}T_{f}}$$

The meaning of each term in the formulas is shown in Table 2:

In the case of DSC, and for the calculation of the areas of specific energy released in the pyrolysis and combustion processes, the NETZSCH software PROTEUS v.8.0.3. has been used.

### 2.4. Mass Spectrometry

To perform the mass spectrometry (MS) analysis, a quadrupole mass spectrometer coupled with the thermobalance, NETSCH model QMS403 Aeolos Quadro (NETZSCH, Selb, Germany), was used. This analysis allows qualitative determination of the volatiles released during the pyrolysis and combustion process of the samples studied. The coupling of the equipment has made it possible to carry out the analyses in series with TG, so that all the tests were conducted in triplicate.

The system has a transfer line linking the thermobalance and the mass spectrometer with a temperature adjustable heated chamber and a precise adjustment of the quartz glass capillary inlet to the spectrometer.

Both the transfer line and the spectrometer are set at a temperature of 200 °C to avoid condensation of the volatile substances in the system. A mass/charge ratio (*m*/*z*) scan was performed to identify the most significant compounds.

### 2.5. Methodology for Statistical Analysis

In order to determine whether the tests performed are reliable, the mean value, the standard deviation (SD), and the relative standard deviation (RSD) of the tests performed in triplicate have been calculated [52,55,56,57].

Also, a statistical analysis of the results of the experiments described above was carried out to determine the influence of the corn crop variety. The objective of the statistical analysis was to determine if the residue from the three types of corn cultivation, that is, conventional, forage, or transgenic, had statistically significant differences between them, based on the results of the proximate and ultimate analysis, TGA and DSC, in order to determine if any particular variety type has better biofuel properties than the other two. For this purpose, the type of corn crop variety was studied as a qualitative variable, and the different values obtained in analysis as quantitative variables, using one-way analysis of variance (ANOVA) to determine the existence of significant differences, and a significance criterion α of 0.05 has been set for the calculation, which represents a confidence interval of 95% [52,56,58]. With ANOVA, two hypotheses are tested, the null hypothesis (H₀), which indicates that there are no significant differences between the means of the groups, and the alternative hypothesis (H_1_), which indicates that at least one mean is significantly different from the others. For the statistical analysis, the R software version 4.2.1 was used in combination with the R Commander package.

This analysis is carried out in three stages, depending on the specific observations of the results. The three stages of the statistical analysis are presented below.

- Stage 1: Normality test

The first step is to check that the data are normally distributed, for which the Shapiro-Wilk test will be applied, since the sample is not large enough to ensure normality [59]. For data that do not follow a normal distribution, the Kruskal-Wallis test can be used as a non-parametric method.

- Stage 2: Levene’s F-test

Levene’s F-test was run to investigate whether the variances in the experimental groups were equal. If Levene’s statistic has a probability of significance greater than 0.05, homoscedasticity of the samples can be considered. In case the Levene’s statistic has a significance probability of less than 0.05, ANOVA together with the Welch F test will be performed without assuming equality of variances.

- Stage 3: Hypothesis setting

**H_0_** **(null hypothesis):** There is no significant difference between the results obtained and the variety of the corn crop.

**H_1_** **(alternative hypothesis):** There are significant differences between the results obtained depending on the type of corn crop variety.

- Stage 4. ANOVA and Kruskal-Wallis test

For normal samples, the ANOVA is performed, otherwise the non-parametric Kruskal-Wallis test is performed. If the probability of significance of the test statistic is above the significance level (α = 0.05), the null hypothesis holds, and no influence by type of corn crop variety is observed. On the contrary, if the significance is lower than 0.05, the null hypothesis is rejected and the influence of the corn crop variety on the results obtained is assumed.

### 2.6. Methodology for Comparative Analysis of Differential Factors Among Corn Varieties

In order to ascertain which studied maize varieties exhibit superior characteristics for pyrolysis and combustion, a comparative analysis of these varieties will be conducted, encompassing all variables considered in the present study.

Regarding the thermochemical analysis, this comparative assessment places particular emphasis on key parameters. The ash content is deemed significant as it is a decisive factor in determining biomass quality. The heating value is also crucial, serving as a parameter that provides insights into the amount of energy released in any thermal process. Additionally, the carbon percentage is considered a significant factor influencing both the heating value and ash content.

As for the thermogravimetric parameters, the primary considerations include the pyrolysis and combustion indices.

Finally, in the case of DSC, the parameter for comparison is the area of energy released in the process.

For the execution of the comparative study, the arithmetic mean value of the 12 corn samples has been obtained. An upper limit is established equal to the mean value plus the standard deviation of the sample, and a lower limit is set equal to the mean value minus the standard deviation of the sample. A positive value is assigned to values exceeding the upper limit, and a negative value to values below the lower limit in each case.

## 3. Results and Discussion

### 3.1. Ultimate and Proximate Analyses and Higher Heating Value

The results concerning the ultimate and proximate analyses, as well as the moisture and higher heating value, can be observed in Table 3.

The moisture content of the 12 residues studied ranged from 7.95% to 10.81%. In all cases, this content was lower than the value recommended by the standard for pelletizing [19]; therefore, pelletizing does not require prior drying of the waste. This aspect is important when the aim is to pelletize the residue for its use in combustion processes.

Different conclusions can be drawn from ultimate analysis results. In all cases, the carbon content was higher than 43%, with the residue of variety C3 having the lowest carbon content with 43.90%. In the case of hydrogen content, the highest value was observed in T3 variety with a value of 5.52%. Regarding nitrogen, the highest value was found in the residues of F1 and F3 varieties, with a value of 0.81%. As for the sulfur content, the values obtained range from 0.05% for sample T1 to 0.27% for sample T2. Focusing on the values obtained for nitrogen and sulfur, as these compounds can produce NO_x_ and SO_2_ emissions, all samples comply with pelletization standard [60] for nitrogen content, which sets a maximum limit of 1.50% for Class A pellets. The same was not valid for sulfur content, with the maximum limit of 0.20% for Class A pellets, being met in all cases except in the case of T2 variety, which means that its use would be limited to the production of Class B pellets. Compared to other herbaceous residues, such as those from rice, bamboo, or potato cultivation, the carbon and hydrogen contents observed are similar [61,62,63,64]. However, compared to other biomasses of wood origin, the carbon and hydrogen content is lower [55,64].

Regarding proximate analysis, specifically ash content, it was observed that the percentage ranged from 5.55% for variety T2 to 8.42% for variety F3. Comparing the ash content with that obtained with other biomasses of herbaceous origin, similar values were observed [45], while in the case of woody biomasses the values were higher [65]. Considering pelletizing standard [60], and the ash contents established in the standard, it was concluded that the varieties F4 and T2 could produce Class A pellets, while the rest of varieties could be used to produce Class B pellets.

In the case of volatile matter, a higher value was observed in the case of transgenic corn varieties, with an average value of 73.09%, while the lowest value was observed in the case of forage corn varieties, with an average value of 72.48%. The observed values were very similar among the different corn groups. Other studies of corn stover have obtained values similar to those reported in the present study [66,67].

As for the calorific value, a value of more than 17 MJ/kg can be observed for all corn varieties, which is considered an optimal value for the use of these residues for their densification. In the case of other herbaceous waste, such as wheat straw, lower values were found [68].

To determine the reliability of the tests, the statistical analysis of the mean and RSD of the parameters performed in triplicate of the proximate and ultimate analysis is shown in Table 4. As can be seen in all cases, the RSD value is lower than 10%, although the RSD value obtained in some cases are slightly higher than the commonly accepted threshold of 5% [52,55,56]. It does not, however, significantly affect the conclusions of the study, as there is literature in which variations of up to 10% are accepted [57]. This calculation could not be performed for parameters where their value is close to zero, i.e., in the case of N and S, since the standard deviation in relation to the mean can produce RSD values that are exaggeratedly high, which distorts the interpretation, and furthermore, according to the statistical analysis below, the result does not affect the rest of the study.

A statistical analysis of the influence of the type of variety, i.e., conventional, forage, or transgenic, on the main values obtained, i.e., moisture content, nitrogen, sulfur, ash, volatile matter, fixed carbon, and calorific value, leads to the following conclusions presented in Table 5.

First of all, the Shapiro-Wilk normality test was carried out, obtaining a significance level higher than 0.05 in all cases, except for the moisture and C content. In this case, the non-parametric Kruskal-Wallis test was required.

As for Levene’s test, equality of variances has been determined in all the cases, except for sulfur content. Therefore, an ANOVA was performed for all the parameters except sulfur, which required Welch’s ANOVA due to the fact that equality of variances could not be determined in the Levene’s test.

Taking all the above into account, it was observed that, in all cases, the null hypothesis was accepted, i.e., it was established that there was no influence of the variety of corn grown. As can be observed, no significant differences were obtained between varieties in terms of N and S content, so that the calculation of the RSD value does not affect the rest of the research carried out.

### 3.2. Thermogravimetric Analysis

#### 3.2.1. Pyrolysis

**Figure 2:**
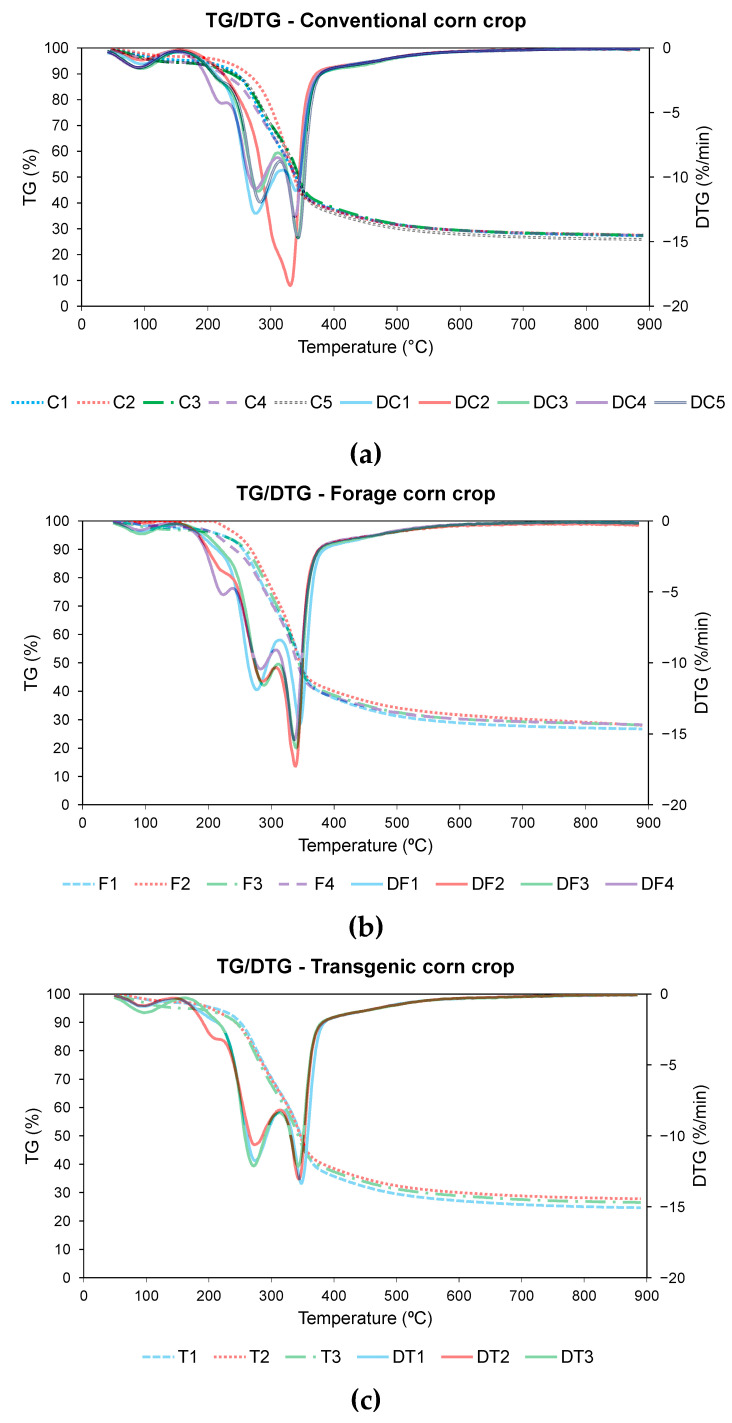
TG and DTG pyrolysis of (**a**) conventional variety, (**b**) forage variety, and (**c**) transgenic variety.

The characteristics inferred from the thermal analysis during pyrolysis are useful to determine the capacity of maintaining self-energy balance in corn residues [18]. Figure 2 shows the TG and DTG profiles of the pyrolysis process for the different varieties of corn, divided according to their typology, that is, conventional, forage, or transgenic. Additionally, Table 6 summarizes the main parameters of the pyrolysis process.

In the pyrolysis curves representative of the pyrolysis process of the 12 samples of corn, a similar thermogravimetric behavior was observed in all cases, with a loss of weight with increasing temperature, due to the loss of moisture and volatilization of compounds during pyrolysis. As in other cases of biomass pyrolysis, three stages can be identified in the pyrolysis process [26,69].

In the first stage of mass loss corresponds to the loss of moisture [69,70,71]. This stage takes place from the start of the test until the temperature T_m_ for the start of fast pyrolysis is reached. According to the data obtained in Table 6, this temperature ranges from 207.3 °C for residue F4 to 274.0 °C for residue C2, which means a wide temperature range among the different corn residues. The lower the pyrolysis start temperature, the better the characteristics of the process, with lower values being observed for residues F4, C4, C1, and T2, without observing a specific trend for each type of corn variety.

The second stage occurs from temperature T_m_ to temperature T_f_, a period in which almost all the mass loss of the pyrolysis process occurs, as well as the decomposition of cellulose and hemicellulose [72,73]. The decomposition of both substances can be seen in the DTG curves, Figure 2, represented by the two peaks where the decomposition rate reaches its maximum value. Li et al. found the same decomposition process in different corn residues during the study of kinetic models [74]. It should be mentioned that in the case of C2 variety the speed of the process was so high that the shoulder peak was not as clearly distinguishable as in the other cases. Another difference to take into account was the case of C1 variety, where the maximum speed peak was reached during the decomposition of the hemicellulose, unlike the rest of the residues where the maximum speed was reached later. The decomposition of lignin in pyrolysis occurs during the process, but without any characteristic peaks [72]. As shown in Figure 2, the maximum peak of hemicellulose decomposition was observed in the range between 271.6 °C for T3 variety and 311.2 °C for C2 variety. In this case, lower temperatures were observed in the case of the transgenic varieties than in the others. On the other hand, the maximum peak of cellulose decomposition oscillates between 330.7 °C for C2 variety and 347.7 °C for T1 variety, with higher values being observed in the case of the transgenic corn. Considering that the lower these temperatures are, the better the pyrolysis process [75], it can be concluded that in the case of transgenic corn, the early onset of hemicellulose decomposition was detrimental to the final pyrolysis process.

Finally, the final carbonization stage is where the decomposition of the biomass occurs, generating a carbonaceous residue as the final product [76]. According to the data obtained in Table 6, this final residue varies between 24.7% for T1 variety and 28.2% for F4 variety, with higher values for the conventional variety. Kumar et al. found similar values for corn stover at different heating rates [77].

In order to compare the characteristics of the different corn varieties in the pyrolysis process, pyrolysis indices were calculated (Table 6). P_i_ and P_f_ indices indicate the ease with which the pyrolysis process is initiated and the pyrolysis yield, while the parameter P is related to the energy required in the process. In all cases, the higher the values of these indices, the better the pyrolysis results. Comparing P_i_ index, lower values were observed for the transgenic variety, with values of 81.94, 81.53 and 74.71%/min^3^ × 10^−3^ for varieties T1, T2, and T3 respectively. This lower value indicates that the pyrolysis process is penalized in this type of waste, while the higher values were obtained for the varieties F4, C2 and F2, with a value of 108.40, 105.60, and 103.65%/min^3^ × 10^−3^, respectively. Comparing P index, values ranging from 1.08%^2^/°C^3^ × 10^−6^ for variety T3 to 1.92%^2^/°C^3^ × 10^−6^ for variety F4 were observed, with a higher mean value in the case of the forage variety. Finally, in the case of P_f_ index, lower values were observed for the transgenic variety, with values of 63.53, 73.18, and 65.18%/min^4^ × 10^−3^ for T1, T2, and T3 varieties, respectively. This lower value indicates that these residues have worse qualities for use in pyrolysis processes.

Jankovic et al. [78] obtained slightly higher values for P index in the study of two biomasses of herbaceous origin, whereas in the study carried out by Paniagua et al. [8] lower values of P index were obtained with biomasses of wood origin.

**Figure 3:**
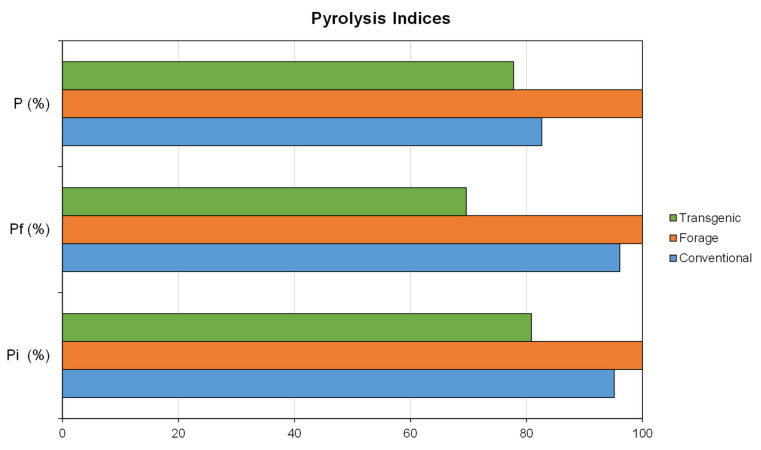
Pyrolysis indices. P_i_ pyrolysis index, P_f_ end pyrolysis index, and P pyrolysis characteristic index.

Comparing the values of P_i_, P, and P_f_ indices, it can be concluded that the transgenic varieties T1, T2, and T3 have worse characteristics for the pyrolysis processes, while the forage varieties have better general characteristics. This can be seen more easily in Figure 3, which compares the relative percentages of the pyrolysis indices.

Table 7 below shows the RSD values of the parameters of the TG tests performed in triplicate, in the pyrolysis process, to determine the reliability of the tests. In all cases, the RSD value is below the 5% threshold [52,55,56].

**Figure 4:**
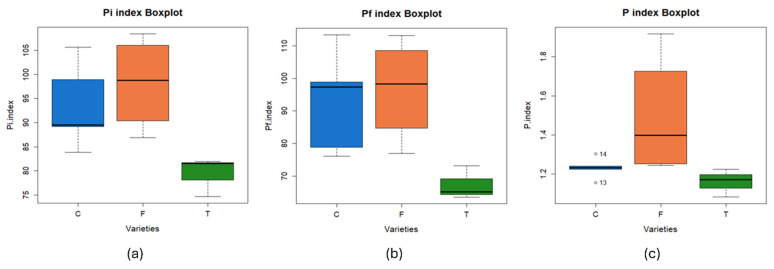
Box plot of the different pyrolysis rates as a function of the type of corn variety grown: (**a**) P_i_ index, (**b**) P_f_ index, and (**c**) P index (C—conventional, F—forage, T—transgenic).

As in the case of ultimate and proximate analyses and higher heating value, a statistical analysis of the influence of the type of variety, i.e., conventional, forage, or transgenic, on the pyrolysis indices obtained has been done. Figure 4 shows the value of the pyrolysis indices in relation to the type of corn grown. Main conclusions of statistical analysis can be observed in Table 8.

Firstly, the Shapiro-Wilk normality test was performed, obtaining a significance level greater than 0.05 in all cases. As for Levene’s test, equality of variances has been determined in the case of P_i_ and P_f_ indices, so an ANOVA has been carried out for these parameters. In the case of the P index, it was necessary to carry out Welch’s ANOVA because it was not possible to establish equality of variances in Levene’s test.

Taking all the above into account, it was observed that the null hypothesis holds, i.e., the influence of the variety of corn grown cannot be determined, for the case of P index, while the null hypothesis was rejected in the cases of P_i_ and P_f_ indices, and it was observed that there was an influence of the variety of corn grown on the value of these indices.

In summary, regarding the pyrolysis process, better characteristics were observed in the forage corn than in the rest of the varieties, with significant differences being observed when comparing P_i_ and P_f_ indices of the different groups.

#### 3.2.2. Combustion

**Figure 5:**
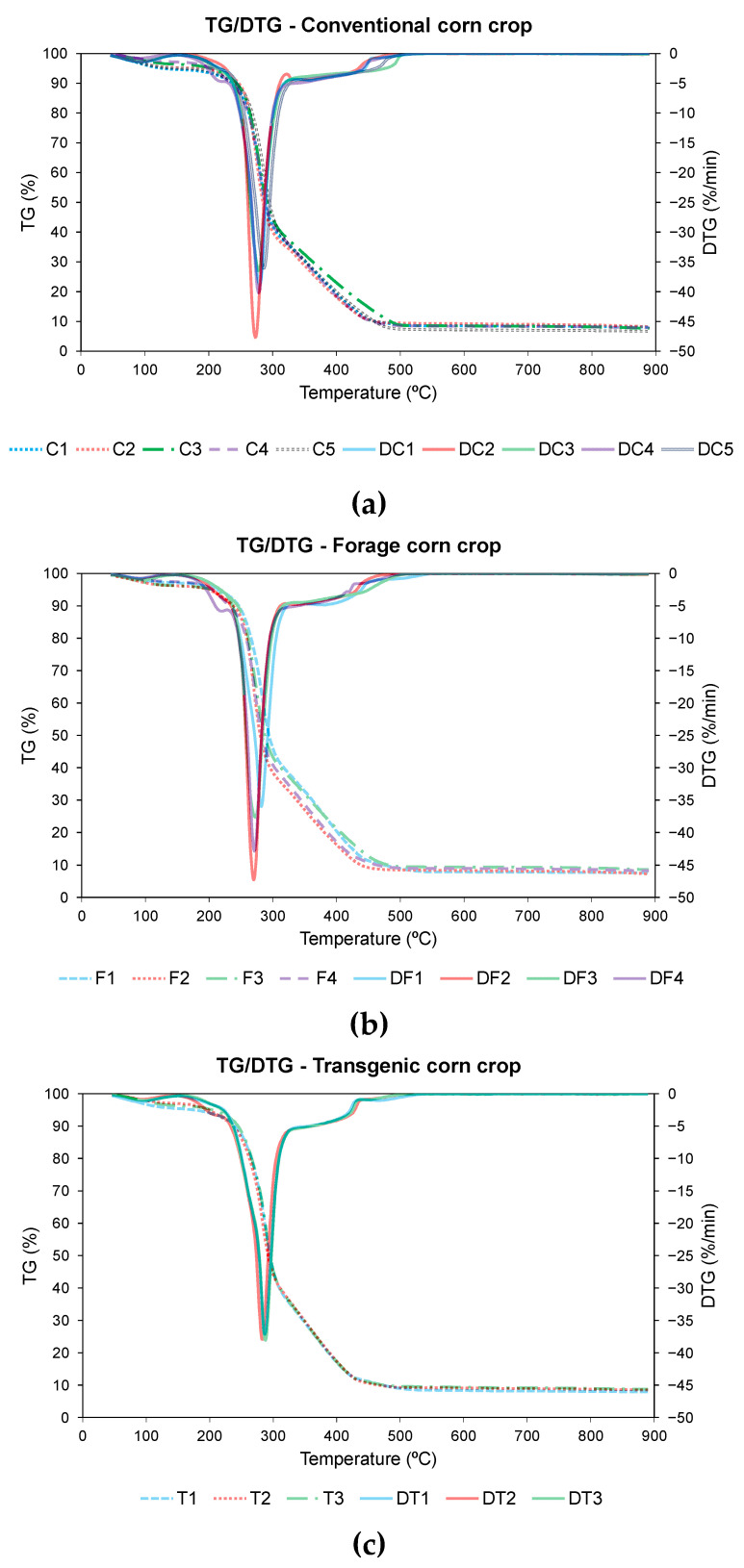
TG and DTG combustion of (**a**) conventional variety, (**b**) forage variety, and (**c**) transgenic variety.

Thermal analysis of the combustion process can be used to compare the characteristics of the different corn varieties in terms of reactivity and combustibility. Figure 5 shows the TG and DTG profiles of the combustion of the different waste. The main combustion parameters of the different corn varieties are included in Table 9.

Analyzing the thermogravimetric graphs, three different stages can be observed during the combustion process in all cases, similar to the behavior obtained with other typologies of biomass [79,80].

The first stage was related to the loss of moisture from the biomass [81]. Comparing the temperature values at which a mass loss of 5% occurs, these values will range from a minimum value of 127 °C for the residue from the conventional variety C1 to a maximum value of 213.0 °C for the residue from the forage variety F1, according to Table 9.

The second stage was related to the combustion of volatile compounds and the decomposition of hemicellulose and cellulose [82]. The temperature at the beginning of this stage is called ignition temperature and it is known that the lower this value is, the better the combustibility properties of the biomass will be [18]. Comparing the values shown in Table 9, there were not many differences in temperature among the 12 samples of corn, obtaining mean values of 253.1 °C for the conventional variety, 248.0 °C for the forage variety and 248.8 °C for the transgenic variety. From Figure 5, it can be seen that the decomposition process of hemicellulose and cellulose occurs together, without compound-specific peaks of maximum speed. In contrast to the pyrolysis process, a single maximum speed peak was observed, with different maximum speeds depending on the corn variety, ranging from a minimum speed of 40.30%/min for the transgenic variety T1 to a maximum speed of 50.40%/min for the forage variety F2. From this point on, the speed decreases until the end of the stage, where there was a change in the slope of the curve TG.

The third stage corresponds to the decomposition of lignin [83], which can be observed as a small plateau at the beginning of this stage and ends at the burnout temperature. The burnout temperature oscillates between 468.6 °C for C1 variety and 510.0 °C for T3 variety. Lignin decomposition was found in the same temperature range for wood biomass [84] and for other herbaceous waste [85].

In order to compare the combustibility properties of each corn variety, the calculation of combustibility indices has been carried out, which are shown in Table 9. D_i_ index indicates the ease with which the combustion process starts while D_f_ index indicates the burnout properties of the biomass, in another way S index indicates the behavior of the combustion process while D index is related to the ease of release of volatile compounds. In all cases, the higher the index value, the better the combustibility properties of the residue.

Comparing D_i_ index, the lowest values were observed in varieties C3 and C5, with values of 280.62 and 265.56%/min^3^ × 10^−3^ respectively, while the highest values were observed in varieties C2 and F2, with values of 376.13 and 388.29%/min^3^ × 10^−3^ respectively. Therefore, there was no clear trend among the different groups of varieties, conventional, forage, and transgenic. Regarding D_f_, no clear trends were observed among the groups either, with lower values for C5 and F1 varieties, with values of 232.52 and 194.54%/min^4^ × 10^−3^ respectively, and higher values for C4 and F2 varieties, with values of 401.34 and 393.66%/min^4^ × 10^−3^, respectively. In the case of D coefficient, lower values were observed for all transgenic varieties, with a value of 3.87, 4.06, and 3.94%/°C^2^ × 10^−3^ for varieties T1, T2, and T3, respectively, while the highest values were observed for varieties C2 and F2, with values of 9.92 and 8.28%/°C^2^ × 10^−3^ respectively. Finally, in the case of S index, the lowest values were observed in the varieties C5 and F1, with values of 31.46 and 31.94%^2^/°C^3^ × 10^−7^ respectively and the highest values in the varieties F2 and F4, with values of 47.34 and 44.74%^2^/°C^3^ × 10^−7^ respectively, with the forage variety being the one with the highest mean value. Mureddu et al. [86] obtained higher S index values for biomasses of wood origin (pine and eucalyptus). On the other hand, Paniagua et al. [52] obtained lower values of S index for a biomass of herbaceous origin, such as quinoa.

**Figure 6:**
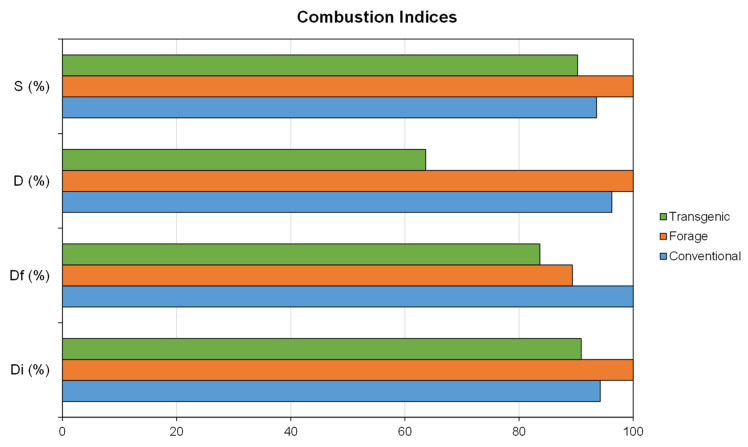
Combustion indices: D_i_ the ignition index, D_f_ burnout index, D devolatilization index, and S combustion characteristic index.

Calculating the relative average values for each variety, as shown in Figure 6, it can be concluded that the best characteristics were observed in the forage variety, while the conventional variety obtains slightly lower values. In the case of the transgenic variety, it was the one that shows the worst characteristics for the combustion process.

Table 10 shows the RSD values of the parameters of the TG tests performed in triplicate, in the combustion process, to determine the reliability of the tests. In the case of combustion, greater variability of the results was observed than in the pyrolysis process, with values of up to 10%, which, as already indicated, is a value accepted in different bibliographies. In the case of DTG_max_ of the C4 and F4 residue, values of over 10% were found, which is within an acceptable range as it is the process of decomposition of cellulose and hemicellulose, in which the process is accelerated in a minimum time, affecting variability.

**Figure 7:**
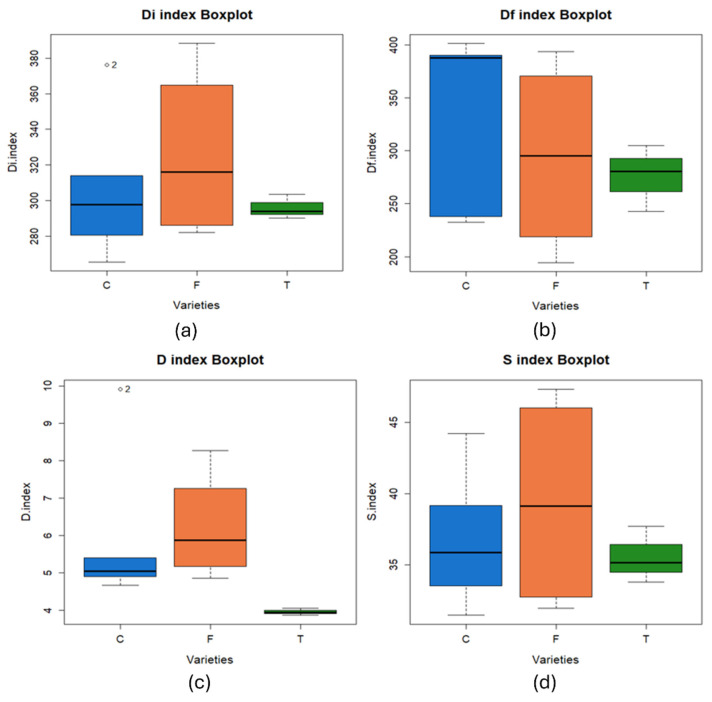
Box plot of the value of the different combustion indices as a function of the type of corn variety grown: (**a**) D_i_ index, (**b**) D_f_ index, (**c**) D index, and (**d**) S index (C—conventional, F—forage, T—transgenic).

A statistical analysis of the influence of the type of variety, i.e., conventional, forage, or transgenic, on the combustion indices obtained has been done. Figure 7 shows the value of the combustion indices in relation to the type of corn grown. Main conclusions of statistical analysis can be observed in Table 11.

After performing the Shapiro-Wilk normality test, a significance level higher than 0.05 was obtained for D_i_ and S indices. As for the Levene’s test, equality of variances was determined only in the case of D_i_ index. Following these results, an ANOVA was performed for D_i_ parameter, the Kruskal-Wallis test for D_f_ and D indices and Welch’s ANOVA for S index.

From the above analysis, it can be noted that the null hypothesis was maintained, i.e., the influence of the variety of maize grown cannot be determined for D_i_, D_f_ and S indices, while the null hypothesis was rejected in the case of D, where it was concluded that there was an influence of the variety of maize grown on the value of this index.

In summary, regarding the combustion process, better characteristics were observed in forage corn than in the rest of the varieties, with significant differences being observed when comparing D index between the separate groups of varieties.

Comparing the pyrolysis and combustion processes, it was observed that pyrolysis was initiated at a slightly lower temperature than combustion, with an average value of 243.67 °C for pyrolysis and 250.32 °C for combustion. The peak temperatures were lower in the case of combustion, with a maximum temperature of 287.10 °C, while in pyrolysis the maximum temperature reached was only 347.70 °C. In the case of pyrolysis, the decomposition of the hemicellulose and cellulose took place in two stages, with two peaks of maximum decomposition rates, while in the combustion process there was only one peak of simultaneous decomposition of the hemicellulose and cellulose. In terms of the duration of the process, the pyrolysis took less time than the combustion, with a minimum time of 4.00 min in the case of the C2 residue compared to 7.40 min for the shortest combustion process with the T1 residue. Mass degradation occurred more quickly in combustion than in pyrolysis, with a DTG_max_ of 50.40%/min for the F2 residue in combustion compared to the DTG_max_ of 18.40%/min for the C2 residue in pyrolysis. This difference is due to the fact that cellulose and hemicellulose decomposed rapidly in combustion, followed by gradual decomposition of lignin [87,88], while in pyrolysis there is no stage of decomposition of lignin [72]. Comparing the residual mass, in the pyrolysis process, it oscillated between 24.65% and 28.08%, higher than in the case of combustion, which oscillated between 6.70% and 8.55%, due to the carbonization produced in the residue during pyrolysis [76]. Finally, greater stability was observed in the pyrolysis process compared to combustion, with less variability in the results due to the rapid decomposition of the cellulosic material during combustion.

### 3.3. Differential Scanning Calorimetry Analysis

**Figure 8:**
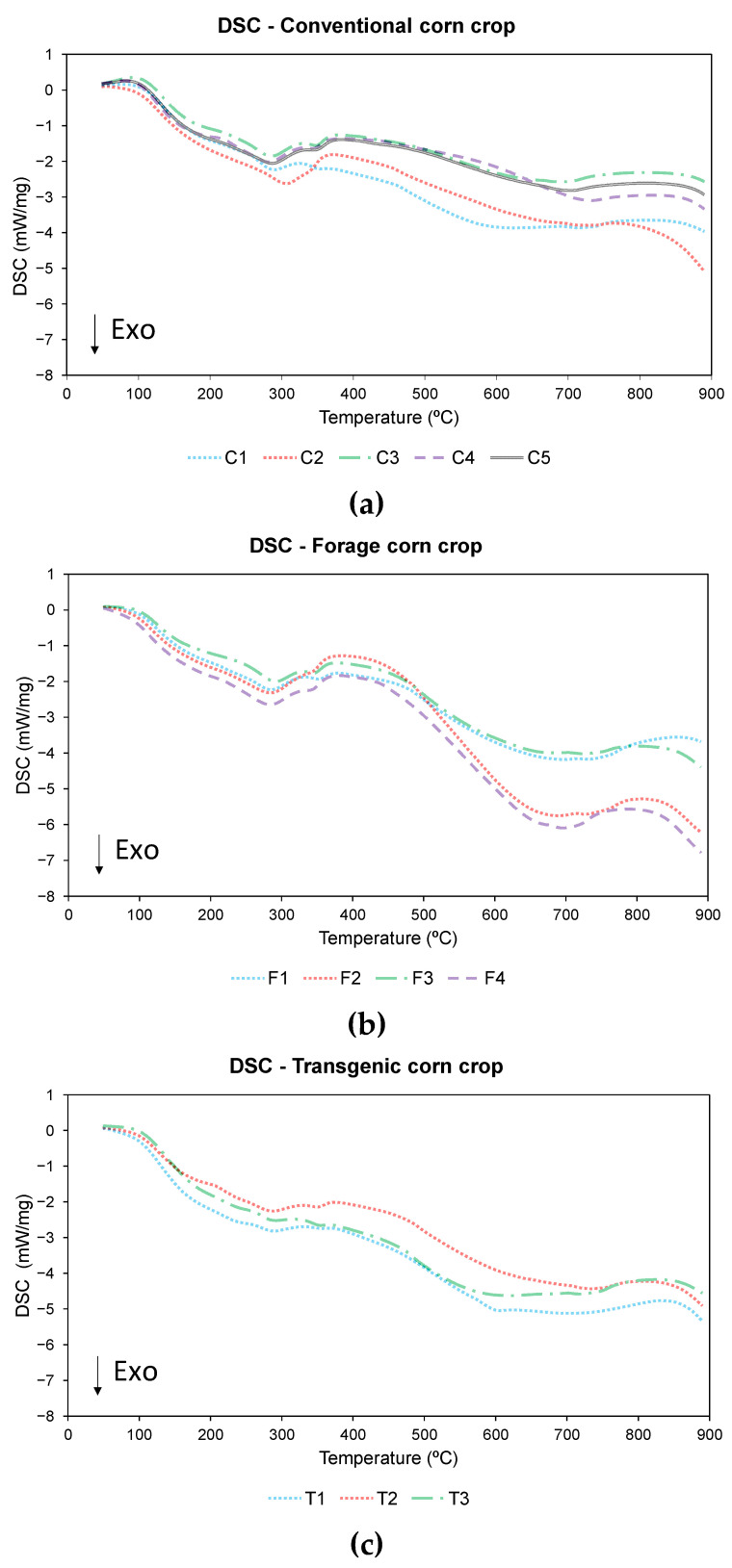
DSC pyrolysis of (**a**) conventional variety, (**b**) forage variety, and (**c**) transgenic variety.

**Figure 9:**
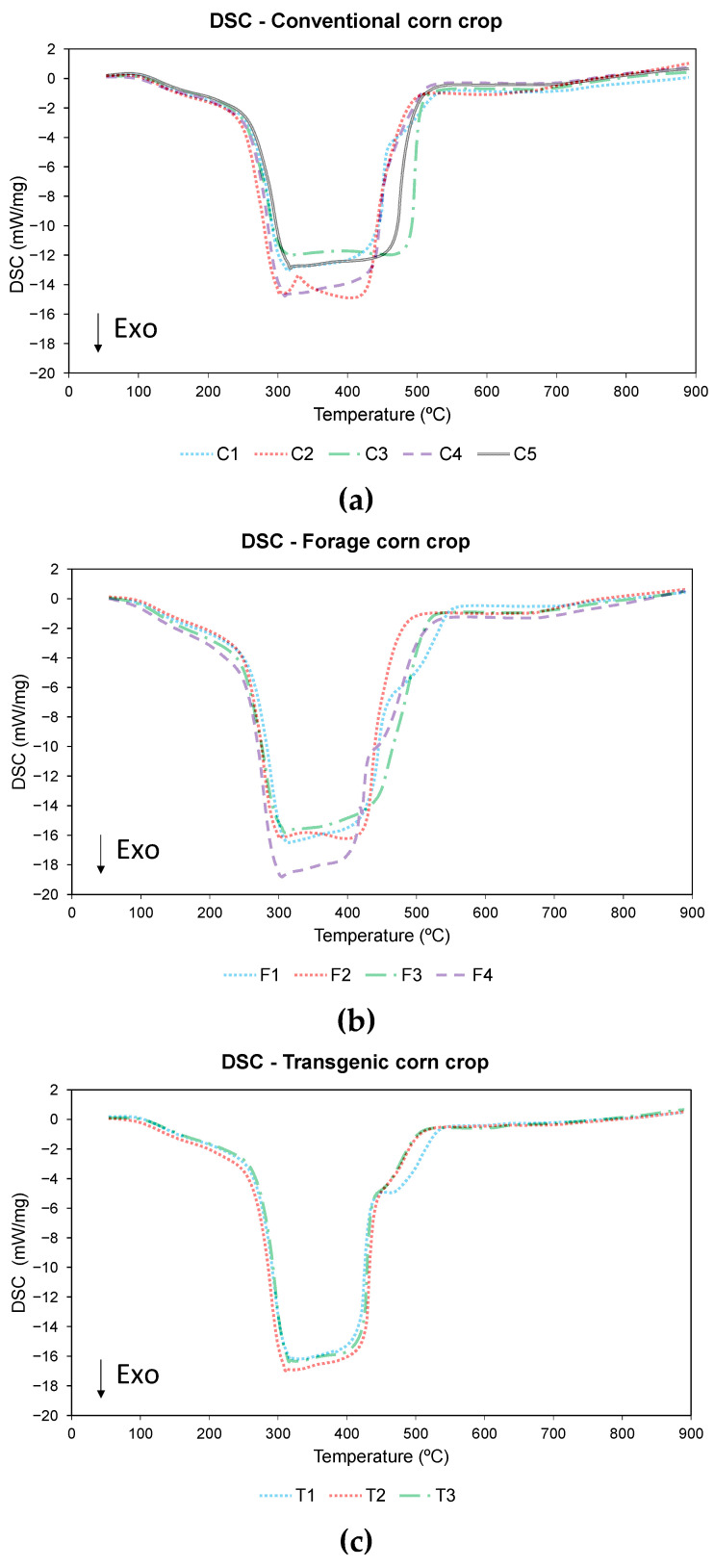
DSC combustion of (**a**) conventional variety, (**b**) forage variety, and (**c**) transgenic variety.

DSC analysis was used to compare the energy behavior of different corn varieties according to the energy consumed in the pyrolysis and combustion processes. The DSC profiles are shown in Figure 8 and Figure 9, for pyrolysis and combustion respectively, while Table 12 shows the values of the exothermic areas obtained in both processes.

During pyrolysis, mainly an exothermic process of energy release was observed without defined minimum peaks. The onset of the process has a small endothermic part due to moisture loss from the sample, which was also observed in other studies [89,90]. After this loss of moisture, the process becomes exothermic in all varieties. This process was mainly exothermic because the percentage of cellulose was higher in biomasses of herbaceous origin than in other woody biomasses [89,91]. During the process, there were two areas where more energy was released, one around 200 °C–300 °C and the other between 600 °C and 700 °C. Comparing the DTG curve with the DSC curve, it can be seen that both processes correspond to the process of decomposition of hemicellulose and cellulose (stage 2 of pyrolysis) and the final stage of carbonization. Using the NETZSCH PROTEUS software v.8.0.3 as a tool, it was observed that the overall exothermic area recorded varies from a minimum value of −636 J/g for variety C1 to a maximum value of −1.932 J/g and −1583 J/g for varieties F1 and F4, respectively.

During combustion, an exothermic process starts around 100 °C and ends around 550 °C in all cases. In this process, a clearly different exothermic area was observed, unlike in the case of pyrolysis process, although there were no differentiated peaks, this behavior was also observed in other studies of biomass combustion processes [92,93,94]. Comparing the DTG curve with the DSC curve, it was observed that the process with the highest energy release occurs between 300 °C and 500 °C, which was approximately the stage at which lignin decomposition takes place, corresponding to stage 3 of the thermogravimetric study. As for the exothermic areas, the minimum value was observed for variety C1 (−7314 J/g), while the maximum value was recorded in varieties F4 (−10,100 J/g) and F3 (−9597 J/g).

In both pyrolysis and combustion processes, it was observed that the C1 variety has the lowest energy release, while the forage varieties have the highest energy value.

To determine the reliability of the DSC test, it was carried out in triplicate and the RSD was then calculated, the values of which are shown in Table 13. The results obtained were below the 10% accepted, taking into account the heterogeneity of the biomass.

As in the previous sections, a statistical analysis was carried out to determine the influence of the corn variety on the values obtained for the DSC areas. Table 14 shows the values obtained from this analysis.

After performing the Shapiro-Wilk normality test and the Levene’s test, a significance level higher than 0.05 was obtained in both cases, so an ANOVA was subsequently performed.

With the results of the above analysis, the null hypothesis was rejected in both cases, and it was therefore concluded that there was an influence of the corn variety grown on the value of the DSC areas obtained.

**Figure 10:**
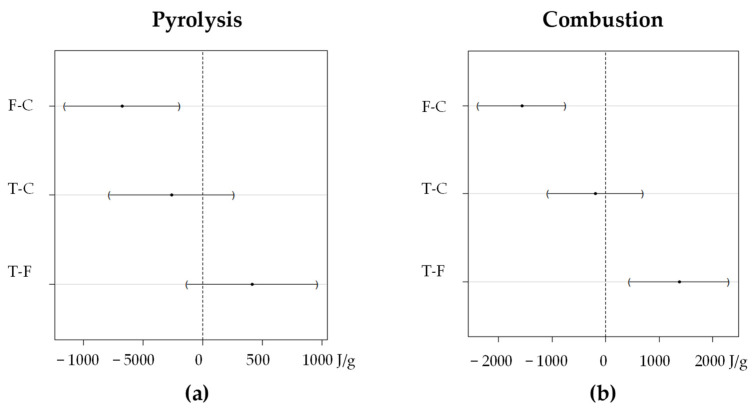
DSC Area, 95% family-wise confidence level in pyrolysis and combustion between corn varieties (**a**) Pyrolysis and (**b**) Combustion.

In the case of the pyrolysis process, a significant difference was observed between the conventional variety and the forage variety, with no differences between the forage and transgenic varieties, or between conventional and transgenic varieties, which can be seen in Figure 10. On the other hand, in the combustion process, significant differences were observed in the forage variety, with no differences between the conventional and transgenic varieties (Figure 10). In both cases, the forage variety shows the highest energy release during the process.

In the case of the combustion process, not only is the exothermic area clearly differentiated, but also a lower variability of the results was found. In the pyrolysis, energy was released practically throughout the test, up to a minimum temperature of 808.00 °C in the case of residue C3, while in combustion it is reduced to a range of temperatures in which the exothermic process occurs, up to a maximum temperature of 575.60 °C in the case of residue F1.

### 3.4. Mass Spectrometry Analysis

**Figure 11:**
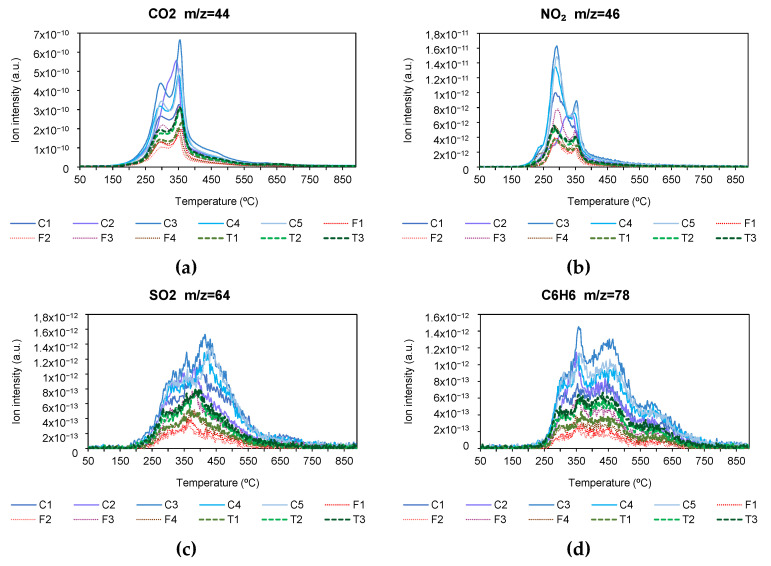
(**a**) CO_2_, (**b**) NO_2_, (**c**) SO_2_, and (**d**) C_6_H_6_ emissions in pyrolysis process (*m*/*z* molecular weight/number of charges).

**Figure 12:**
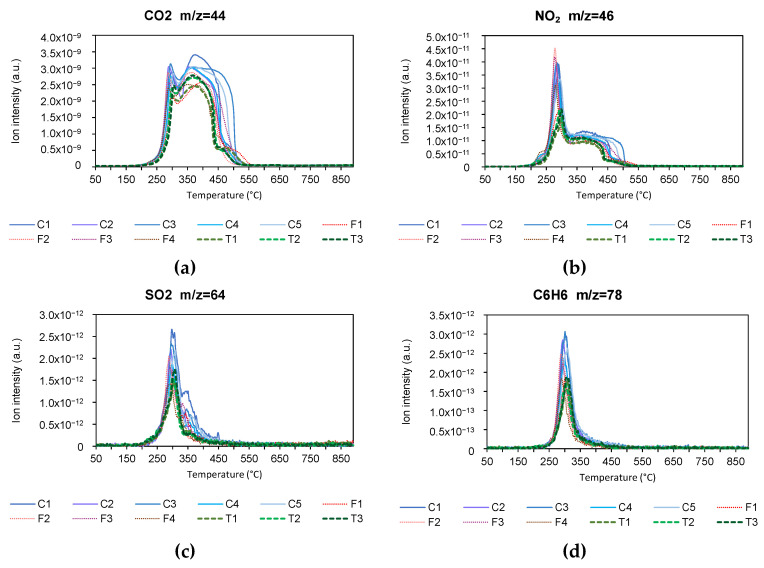
(**a**) CO_2_, (**b**) NO_2_, (**c**) SO_2_, and (**d**) C_6_H_6_ emissions in combustion process (*m*/*z* molecular weight/number of charges).

The results of the emissions measured with the mass spectrometer for the pyrolysis and combustion process are shown below. Figure 11 and Figure 12 show the emissions of CO_2_, NO_2_, SO_2_, and C_6_H_6_, in pyrolysis and combustion, respectively. CO_2_ emissions are shown as it was a very important compound in both pyrolysis and combustion. Likewise, NO_2_ and SO_2_ are considered important because of their ability to form NOx and SOx compounds. Finally, the compound C_6_H_6_ was studied because of the possible adverse effects of its emission.

During the pyrolysis process, CO_2_ emissions were mainly produced between 250 °C and 450 °C, corresponding to stage 2 of the volatilization process, and it was also observed that the intensity of emissions was higher in conventional varieties, followed by the transgenic variety and finally the forage variety. Similar behavior was observed in the NO_2_ emissions graph. With regard to the SO_2_ and C_6_H_6_ emissions graph, it can be seen that the process extends to higher temperatures, including stages 2 and 3 of the pyrolysis process, with a higher intensity of emissions in the conventional variety, followed by the transgenic variety and finally the forage variety. Tang S. et al. [95] obtained similar results in the pyrolysis of corn stover, whereas Maurya R. et al. [96] also obtained similar results for other biomass products.

During the combustion process, CO_2_ emissions occur between 250 °C and 450 °C, covering the entire decomposition stage of hemicellulose, cellulose, and lignin. The intensity of emissions was higher in the conventional variety, while there were no differences between the forage and transgenic varieties.

As for NO_2_ emissions, these also occur between 250 °C and 450 °C, but in this case, a peak related to the decomposition of hemicellulose and cellulose was observed between 250 °C and 350 °C, and a plateau related to the decomposition of lignin at a temperature between 350 °C and 450 °C, with a higher intensity of emissions in all conventional varieties and a lot of variability in the forage variety.

In the case of SO_2_ and C_6_H_6_, emissions were concentrated between 250 °C and 350 °C, i.e., in stage 2 of combustion, with a higher emission intensity for conventional varieties and similar between transgenic and forage varieties. Similar behavior has been observed for other biomass products [84,97].

### 3.5. Comparative Analysis of Differential Factors Among Corn Varieties

For the comparative analysis of the different study varieties in this research, values for the most relevant parameters were extracted and the mean, upper limit, and lower limit were calculated. As previously explained, a positive point was assigned to values exceeding the upper limit, and a negative point to values below the lower limit in each case. The ash content and carbon percentage from the proximate and ultimate analyses, the higher heating value, the pyrolysis and combustion indices from TG, and finally, the exothermic areas from DSC have been identified as the most decisive parameters. All the parameters chosen in this comparison were considered the most relevant from the global perspective of the use of the waste as biofuel, taking into account its thermal characteristics.

**Figure 13:**
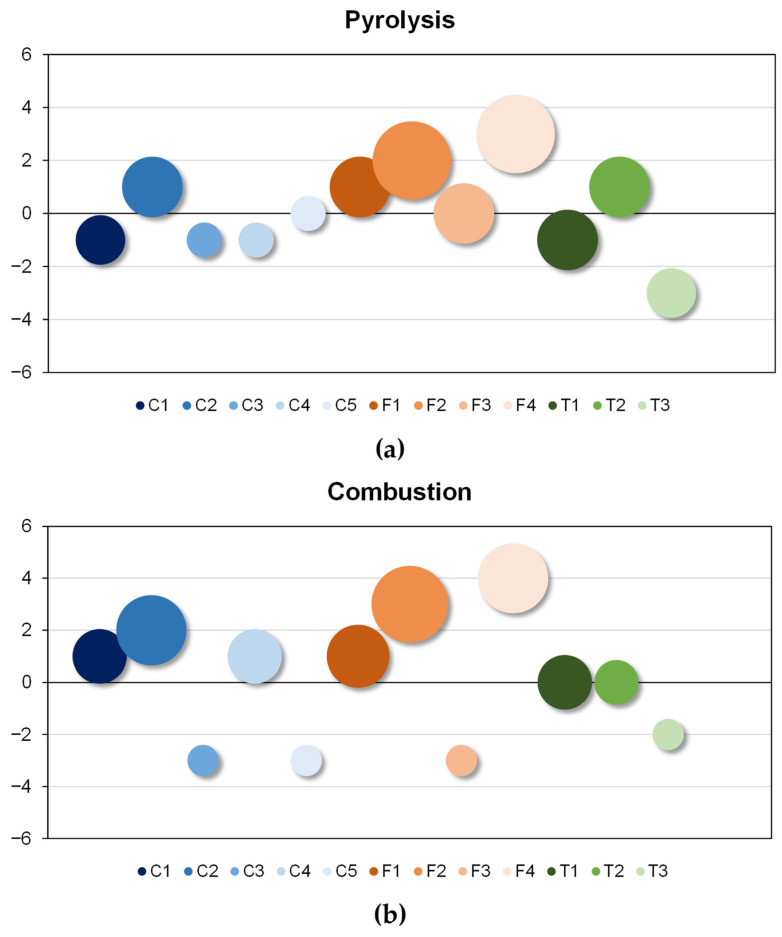
(**a**) Pyrolysis and (**b**) Combustion comparative analyses.

The obtained data has been graphically represented (Figure 13). The optimal quality of each variety was determined by its position on the vertical axis—higher values indicating better characteristics—and the size of the corresponding symbol (dot diameter)-larger sizes represent a greater number of positive parameters.

Generally, in both cases, it was observed that the forage variety exhibits superior qualities, both in position and the size of the representative icon. However, within the same variety, during the combustion process, one of the samples differs from the rest of the samples of the same variety. On the contrary, the variety exhibiting inferior characteristics was the transgenic variant, particularly in combustion processes. It is noteworthy that in both processes, pyrolysis and combustion, the forage variety F4 exhibits the most favorable characteristics for use as a biofuel. The overall study of the thermal characteristics investigated in the three categories of corn residues, makes forage corn residues the most suitable for use as biofuel.

Comparing the results obtained in pyrolysis and combustion, a better homogeneity of results was observed in this comparative analysis in the pyrolysis process than in the combustion process, especially for the conventional and forage varieties. This result was also observed in the comparison of the results of the TG, which, as already indicated, is related to the rate of decomposition of matter.

The advantageous qualities of forage corn waste for use as biofuel were considered a very important aspect, since its cultivation can provide a competitive advantage when it comes to making the waste generated by the crop profitable, since traditionally the market price of the product produced from this variety of corn was lower than that of the other varieties studied.

In addition, it should be noted that the cultivation of one variety or another does not involve any additional economic or logistical considerations and its application would not have any impact. In general, for any variety of corn, the collection of residues for subsequent energy use involves an economic cost and associated logistics, which is common to all agroforestry residues as a whole, so it will not constitute a significant differential between the different varieties for their subsequent utilization.

## 4. Conclusions

This study was dedicated to determining the characteristics of 12 different corn samples, categorized into three groups of varieties, as biofuels. A thorough examination of various quality parameters of biomass was conducted through thermo-chemical analysis and TG/DSC/EM analyses. Overall, it was observed that all varieties exhibit optimal values for use as biofuels in both pyrolysis and combustion processes.

Regarding proximate and ultimate analyses, notable characteristics such as an adequate carbon content, higher than 43.9%, elevated volatile content, higher than 71.01%, and a low ash percentage, lower than 8.5% in all cases, were observed, with these parameters being determinants in evaluating the viability of a solid biofuel, and specifically for subsequent domestic use after pelletizing. As for TG, significant differences were observed among the three varieties, highlighting superior qualities in the forage variety, evident in both the pyrolysis and combustion processes. Additionally, significant differences were observed in the results of the DSC analysis, further underscoring the superior attributes of the forage variety compared to the other two varieties.

In general, superior characteristics of the forage variety were noted in comparison to the conventional and transgenic varieties, in both pyrolysis and combustion processes, with significant differences observed between the three varieties, which can be a competitive advantage for this type of forage corn crop.

In conclusion, this article serves to demonstrate that agricultural crop residues, such as corn, were suitable for use as biofuels. Additionally, the study highlights that the yield may vary depending on the variety of corn cultivated.

## Figures and Tables

**Table 1:** Different pyrolysis terms.

Term	
DTG_max_ (%/min)	maximum pyrolysis rate
DTG_mean_ (%/min)	mean pyrolysis rate
T_m_ (°C)	starting temperature
T_f_ (°C)	end temperature
t_m_ (min)	corresponding time of the starting temperature
t_p_ (min)	corresponding time of the maximum pyrolysis rate
t_f_ (min)	corresponding time of the end temperature
Δt_(1/2) (min)	time range that corresponds to *DTG*/*DTG_max_* = ½

**Table 2:** Different combustion terms.

Term	
DTG_max_ (%/min)	maximum combustion rate
DTG_mean_ (%/min)	mean combustion rate
T_i_ (°C)	ignition temperature
T_p_ (°C)	peak temperature
T_f_ (°C)	burnout temperature
ΔT (°C)	difference between T_p_ and T_i_
t_i_ (min)	ignition time
t_p_ (min)	corresponding time of the maximum combustion rate
t_f_ (min)	burnout time
Δt_(1/2) (min)	time range that corresponds to DTG/DTG_max_ = ½

**Table 3:** Ultimate and proximate analyses and heating value (db: dry basis, wb: wet basis).

Variety	Moisture (% wb)	Ultimate Analysis (% db)					Proximate Analysis (% db)			HHV (MJ/kg db)
		C	H	N	S	O	Ash	Volatile Matter	Fixed Carbon	
C1	8.03	44.70 ± 0.32	5.19 ± 0.10	0.27 ± 0.03	0.08 ± 0.02	49.76	6.12 ± 0.20	73.94 ± 0.24	19.94	17.38 ± 0.30
C2	9.29	44.10 ± 0.04	5.29 ± 0.02	0.22 ± 0.03	0.1 ± 0.02	50.29	6.67 ± 0.66	74.36 ± 0.45	18.97	17.09 ± 0.35
C3	8.67	43.90 ± 0.63	5.04 ± 0.16	0.65 ± 0.28	0.07 ± 0.01	50.34	7.14 ± 0.40	71.49 ± 0.19	21.37	17.65 ± 0.08
C4	8.88	45.20 ± 0.57	5.07 ± 0.07	0.52 ± 0.50	0.1 ± 0.01	49.11	7.61 ± 0.61	71.01 ± 0.38	21.38	17.66 ± 0.06
C5	9.13	44.70 ± 0.03	5.15 ± 0.09	0.47 ± 0.08	0.15 ± 0.08	49.53	7.51 ± 0.39	72.74 ± 0.44	19.75	17.37 ± 0.51
F1	7.96	45.30 ± 0.33	5.49 ± 0.03	0.81 ± 0.10	0.15 ± 0.02	48.25	7.49 ± 0.32	72.16 ± 0.12	20.35	17.93 ± 0.10
F2	7.95	44.30 ± 0.30	5.46 ± 0.17	0.56 ± 0.07	0.11 ± 0.01	49.57	8.40 ± 0.46	72.72 ± 0.44	18.88	17.56 ± 0.02
F3	10.39	44.20 ± 0.26	5.37 ± 0.16	0.81 ± 0.02	0.19 ± 0.02	49.43	8.42 ± 0.27	71.71 ± 0.04	19.87	17.06 ± 0.57
F4	8.14	44.70 ± 0.42	5.39 ± 0.13	0.34 ± 0.05	0.12 ± 0.01	49.45	5.95 ± 0.38	73.31 ± 0.35	20.74	17.60 ± 0.06
T1	10.75	45.20 ± 0.15	5.42 ± 0.01	0.61 ± 0.05	0.05 ± 0.01	48.72	8.27 ± 0.62	72.14 ± 0.30	19.59	17.80 ± 0.15
T2	10.16	45.00 ± 0.08	5.49 ± 0.03	0.49 ± 0.23	0.27 ± 0.04	48.75	5.55 ± 0.25	72.29 ± 0.22	22.16	17.56 ± 0.23
T3	10.81	45.00 ± 0.20	5.52 ± 0.04	0.32 ± 0.21	0.08 ± 0.02	49.08	7.17 ± 0.12	74.85 ± 5.71	17.98	17.25 ± 0.26

**Table 4:** Results of RSD of ultimate and proximate analyses and heating value tests.

Variety	Ultimate Analysis (% db)		Proximate Analysis (% db)		HHV
	C	H	Ash	Volatile Matter	(MJ/kg db)
C1	0.72	1.93	3.29	0.33	1.71
C2	0.09	0.38	9.82	0.61	2.05
C3	1.44	3.17	5.61	0.26	0.45
C4	1.26	1.38	8.08	0.53	0.31
C5	0.07	1.75	5.14	0.61	2.92
F1	0.73	0.55	4.21	0.17	0.54
F2	0.68	3.11	5.47	0.60	0.11
F3	0.59	2.98	3.22	0.05	3.35
F4	0.94	2.41	6.37	0.48	0.32
T1	0.33	0.00	7.49	0.41	0.85
T2	0.18	0.55	4.42	0.30	1.31
T3	0.44	0.72	1.70	7.62	1.52

**Table 5:** Results of the statistical analysis in ultimate and proximate analyses and heating value tests.

Parameter	Shapiro-Wilk	Levene’s	Test	*p*-Value	Conclusion
% Moisture	Non-normal	Equal	Kruskal-Wallis	0.056	Retain the null hypothesis
% C	Non-normal	Equal	Kruskal-Wallis	0.291	Retain the null hypothesis
% N	Normal	Equal	ANOVA	0.309	Retain the null hypothesis
% S	Normal	Unequal	ANOVA	0.316	Retain the null hypothesis
% Ash	Normal	Equal	ANOVA	0.679	Retain the null hypothesis
% Volatile matter	Normal	Equal	ANOVA	0.821	Retain the null hypothesis
% CF	Normal	Equal	ANOVA	0.904	Retain the null hypothesis
PCS (MJ/kg)	Normal	Equal	ANOVA	0.824	Retain the null hypothesis

**Table 6:** Main pyrolysis parameters (inert atmosphere).

	Main Pyrolysis Parameters											
	Conventional					Forage				Transgenic		
	C1	C2	C3	C4	C5	F1	F2	F3	F4	T1	T2	T3
Pyrolysis initial T_m_ (°C)	239.0 ± 0.7	274.0 ± 0.2	247.9 ± 0.3	235.8 ± 0.1	248.2 ± 3.1	244.3 ± 0.2	245.6 ± 1.8	259.0 ± 1.7	207.3 ± 1.8	245.8 ± 1.2	235.5 ± 0.6	241.6 ± 2.6
Peak temperature T_p_ (°C)	276.0 ± 0.4	330.7 ± 0.6	342.8 ± 0.6	338.3 ± 0.4	343.1 ± 0.6	345.2 ± 1.6	338.0 ± 0.6	339.7 ± 0.5	336.9 ± 0.2	347.7 ± 0.2	343.9 ± 0.7	342.9 ± 0.1
Shoulder peak T_s_ (°C)	339.8 ± 0.3	311.2 ± 2.0	280.1 ± 0.8	276.1 ± 0.1	283.1 ± 1.1	277.2 ± 0.6	286.9 ± 3.8	288.3 ± 2.1	283.3 ± 0.4	274.2 ± 0.2	273.0 ± 2.1	271.6 ± 1.2
Pyrolysis finish T_f_ (°C)	382.6 ± 1.2	366.2 ± 0.6	376.7 ± 0.7	378.6 ± 1.2	380.9 ± 1.8	383.5 ± 1.5	369.5 ± 1.4	373.4 ± 1.9	372.4 ± 2.6	387.3 ± 1.1	383.1 ± 1.4	384.4 ± 1.9
DTG peak max (%/min)	12.8 ± 0.2	18.4 ± 0.3	14.7 ± 0.1	13.1 ± 0.1	14.7 ± 0.2	14.4 ± 0.3	16.9 ± 0.3	16.0 ± 0.2	15.4 ± 0.4	13.4 ± 0.3	13.1 ± 0.1	12.1 ± 0.2
DTG shoulder (%/min)	11.1 ± 0.3	15.9 ± 0.5	11.1 ± 0.2	10.9 ± 0.2	11.9 ± 0.3	11.9 ± 0.2	11.1 ± 0.4	11.6 ± 0.5	10.4 ± 0.1	11.8 ± 0.1	10.6 ± 0.2	12.1 ± 0.2
DTG mean (%/min)	2.0	1.9	1.9	2.0	2.0	2.0		2.0		2.0	2.0	
Pyrolysis initial t_m_ (min)	10.7 ± 0.0	12.1 ± 0.2	11.0 ± 0.1	10.6 ± 0.1	11.0 ± 0.1	11.0 ± 0.0	11.0 ± 0.2	11.5 ± 0.1	9.7 ± 0.1	10.8 ± 0.0	10.7 ± 0.0	10.8 ± 0.1
Peak time t_p_ (min)	12.1 ± 0.0	14.4 ± 0.1	14.9 ± 0.0	14.7 ± 0.1	15.0 ± 0.0	15.1 ± 0.2	14.8 ± 0.1	14.8 ± 0.0	14.7 ± 0.0	15.0 ± 0.1	15.0 ± 0.1	15.0 ± 0.0
Pyrolysis finish t_f_ (min)	16.8 ± 0.1	16.1 ± 0.1	16.6 ± 0.6	16.7 ± 0.1	16.8 ± 0.1	17.0 ± 0.1	16.3 ± 0.1	16.5 ± 0.1	16.4 ± 0.1	17.1 ± 0.1	17.0 ± 0.1	17.0 ± 0.1
P_i_ (%/min^3^ × 10^−3^)	98.94	105.60	89.51	83.88	89.21	86.87	103.65	93.90	108.40	81.94	81.53	74.71
P_f_ (%/min^4^ × 10^−3^)	78.77	113.38	98.85	76.06	97.35	76.92	113.18	104.05	92.52	63.53	73.18	65.18
P (%^2^/°C^3^ × 10^−6^)	1.15	1.30	1.23	1.22	1.24	1.26	1.54	1.24	1.92	1.17	1.22	1.08
Residual mass (%)	27.3 ± 0.6	27.5 ± 1.2	27.6 ± 0.1	27.4 ± 0.2	25.8 ± 0.1	26.7 ± 0.7	27.7 ± 0.2	28.1 ± 0.8	28.2 ± 0.4	24.7 ± 0.8	27.8 ± 0.4	26.5 ± 0.3

**Table 7:** Results of RSD of pyrolysis TG.

	Main Pyrolysis Parameters											
	Conventional					Forage				Transgenic		
	C1	C2	C3	C4	C5	F1	F2	F3	F4	T1	T2	T3
Pyrolysis initial T_m_ (°C)	0.28	0.07	0.10	0.05	1.23	0.09	0.34	0.64	0.88	0.47	0.26	1.05
Peak temperature T_p_ (°C)	015	0.18	0.17	0.10	0.16	0.48	0.19	0.13	0.06	0.04	0.19	0.02
Shoulder peak T_s_ (°C)	0.08	0.65	0.29	0.02	0.39	0.21	1.33	0.73	0.14	0.06	0.74	0.43
Pyrolysis finish T_f_ (°C)	0.30	0.17	0.18	0.30	0.48	0.39	0.37	0.51	0.71	0.29	0.36	0.48
DTG peak max (%/min)	1.34	1.40	0.92	0.92	1.53	2.32	1.95	1.02	2.53	2.02	0.62	1.51
DTG shoulder (%/min)	2.17	2.87	2.14	2.11	2.15	1.94	3.18	3.79	1.02	0.89	1.99	1.79
Pyrolysis initial t_m_ (min)	0.00	1.28	0.52	0.54	0.90	0.37	1.42	0.48	0.77	0.32	0.27	0.74
Peak time t_p_ (min)	0.00	0.81	0.00	0.39	0.00	1.12	0.55	0.08	0.04	0.65	0.34	0.04
Pyrolysis finish t_f_ (min)	0.34	0.63	3.91	0.35	0.60	0.81	0.89	0.56	0.78	0.29	0.45	0.65
Residual mass (%)	2.28	4.55	0.31	0.60	0.43	2.58	4.44	0.25	1.34	3.29	1.59	0.93

**Table 8:** Results of the statistical analysis in pyrolysis indices.

Parameter	Shapiro-Wilk	Levene’s	Test	*p*-Value	Conclusion
P_i_	Normal	Equal	ANOVA	0.041	Reject the null hypothesis
P_f_	Normal	Equal	ANOVA	0.047	Reject the null hypothesis
P	Normal	Unequal	Welch’s ANOVA	0.210	Retain the null hypothesis

**Table 9:** Main combustion parameters (air atmosphere).

	Main Combustion Parameters											
	Conventional					Forage				Transgenic		
	C1	C2	C3	C4	C5	F1	F2	F3	F4	T1	T2	T3
T 5% weigh loss (°C)	127.0	182.0	203.0	204.0	152.0	213.0	199.0	206.0	206.0	176.0	199.0	205.0
Ignition temp. T_i_ (°C)	253.2 ± 0.5	254.7 ± 2.2	250.9 ± 2.4	248.3 ± 2.0	258.4 ± 2.8	252.8 ± 1.9	248.1 ± 2.7	251.0 ± 1.1	239.9 ± 6.6	249.7 ± 0.4	246.1 ± 1.4	250.7 ± 3.2
Peak temp. T_p_ (°C)	280.1 ± 0.8	273.2 ± 1.2	278.8 ± 1.3	278.6 ± 2.2	286.5 ± 1.8	281.3 ± 4.3	270.6 ± 1.1	273.7 ± 0.5	270.5 ± 1.6	286.1 ± 0.3	282.0 ± 0.6	287.1 ± 1.6
Burnout temp. T_f_ (°C)	468.6 ± 7.3	471.0 ± 29.7	482.0 ± 2.6	474.0 ± 12.2	475.0 ± 6.0	504.0 ± 20.1	473.0 ± 7.1	486.0 ± 44.3	479.0 ± 5.4	485.0 ± 5.8	478.0 ± 9.0	510.0 ± 20.7
DTG_max_ (%/min)	40.7 ± 1.0	50.1 ± 4.1	38.0 ± 1.7	42.5 ± 6.0	37.5 ± 3.4	38.9 ± 2.3	50.4 ± 1.7	38.7 ± 3.3	45.5 ± 5.7	40.3 ± 1.9	41.1 ± 1.2	41.2 ± 2.5
DTG_mean_ (%/min)	2.7	2.7	2.7	2.7	2.7	2.7	2.7	2.7	2.7	2.6	2.7	2.6
Ignition time t_i_ (min)	11.2 ± 0.1	11.2 ± 0.1	11.2 ± 0.1	11.1 ± 0.1	11.4 ± 0.1	11.2 ± 0.1	11.0 ± 0.1	11.2 ± 0.0	11.2 ± 0.2	11.2 ± 0.1	11.0 ± 0.1	11.2 ± 0.1
Peak time t_p_ (min)	12.2 ± 0.0	11.9 ± 0.1	12.1 ± 0.1	12.2 ± 0.1	12.4 ± 0.1	12.3 ± 0.1	11.8 ± 0.1	11.9 ± 0.0	11.9 ± 0.1	12.4 ± 0.1	12.3 ± 0.1	12.5 ± 0.1
Burnout time t_f_ (min)	21.5 ± 0.4	21.6 ± 1.6	22.0 ± 0.1	21.7 ± 0.6	21.7 ± 0.3	23.2 ± 1.0	21.7 ± 0.4	22.3 ± 0.1	22.0 ± 0.3	22.3 ± 0.3	21.9 ± 0.4	23.5 ± 1.1
D_i_ (%/min^3^ × 10^−3^)	297.64	376.13	280.62	313.84	265.56	282.08	388.29	290.29	341.54	290.18	303.47	294.07
S (%^2^/°C^3^ × 10^−7^)	35.85	44.24	33.52	39.16	31.46	31.94	47.34	33.51	44.74	35.12	37.70	33.80
D (%/°C^2^ × 10^−3^)	5.40	9.92	4.89	5.03	4.66	4.85	8.28	6.23	5.50	3.87	4.06	3.94
D_f_ (%/min^4^ × 10^−3^)	387.63	390.06	238.10	401.34	232.52	194.54	393.66	242.99	347.75	242.90	304.86	280.31
Residual mass (%)	8.1 ± 0.1	8.1 ± 0.8	7.6 ± 0.1	7.9 ± 0.3	6.7 ± 0.3	7.6 ± 0.7	7.3 ± 0.7	8.5 ± 0.2	8.4 ± 0.9	7.9 ± 0.8	8.6 ± 0.3	8.8 ± 0.7

**Table 10:** Results of RSD of combustion TG.

	Main Combustion Parameters											
	Conventional					Forage				Transgenic		
	C1	C2	C3	C4	C5	F1	F2	F3	F4	T1	T2	T3
Ignition temp. T_i_ (°C)	0.18	0.84	0.95	0.81	1.09	0.76	1.06	0.42	2.67	0.16	0.57	1.24
Peak temp. T_p_ (°C)	0.28	0.42	0.45	0.79	0.62	1.53	0.39	0.19	0.59	0.10	0.20	0.56
Burnout temp. T_f_ (°C)	1.56	6.79	0.55	2.63	1.27	4.17	1.53	9.63	1.15	1.20	1.92	4.24
DTG_max_ (%/min)	2.47	8.16	4.32	14.05	8.41	5.92	3.38	7.88	12.01	4.70	3.05	6.54
Ignition time t_i_ (min)	0.51	0.88	0.51	0.90	1.00	1.02	0.9	0.00	1.89	1.05	0.52	0.51
Peak time t_p_ (min)	0.00	0.49	0.48	0.47	0.47	0.47	0.49	0.00	0.84	0.81	0.47	0.81
Burnout time t_f_ (min)	1.77	8.21	0.53	2.86	1.24	4.36	1.65	0.52	1.22	1.46	1.88	4.74
Residual mass (%)	1.28	9.96	1.55	4.11	4.63	9.97	8.51	1.79	9.99	9.92	3.38	8.14

**Table 11:** Results of the statistical analysis in combustion indices.

Parameter	Shapiro-Wilk	Levene’s	Test	*p*-Value	Conclusion
D_i_	Normal	Equal	ANOVA	0.626	Retain the null hypothesis
D_f_	Non-normal	Unequal	Kruskal-Wallis	0.832	Retain the null hypothesis
D	Non-normal	Unequal	Kruskal-Wallis	0.038	Reject the null hypothesis
S	Normal	Unequal	Welch’s ANOVA	0.648	Retain the null hypothesis

**Table 12:** Area DSC pyrolysis and combustion (Negative values correspond to exothermic behavior).

	Area DSC											
	Conventional					Forage				Transgenic		
	C1	C2	C3	C4	C5	F1	F2	F3	F4	T1	T2	T3
Pyrolysis Area (J/g)	−636 ± 40	−1248 ± 121	−910 ± 86	−931 ± 77	−981 ± 52	−1932 ± 127	−1784 ± 152	−1176 ± 22	−1583 ± 95	−1355 ± 103	−980 ± 93	−1275 ± 99
Combustion Area (J/g)	−7314 ± 275	−8317 ± 239	−8042 ± 159	−8126 ± 10	−7715 ± 105	−9470 ± 142	−8707 ± 112	−9597 ± 538	−10,100 ± 339	−8131 ± 119	−8270 ± 48	−7873 ± 316

**Table 13:** Results of RSD of DSC.

	Area DSC											
	Conventional					Forage				Transgenic		
	C1	C2	C3	C4	C5	F1	F2	F3	F4	T1	T2	T3
Pyrolysis	6.15	8.81	8.71	8.57	5.35	6.58	9.47	1.87	6.46	8.29	8.67	836
Combustion	3.67	2.79	1.99	0.13	1.36	1.52	1.31	5.94	3.39	1.49	0.58	4.21

**Table 14:** Results of the statistical analysis DSC Area.

Parameter	Shapiro-Wilk	Levene’s	Test	*p*-Value	Conclusion
Pyrolysis Area	Normal	Equal	ANOVA	0.002	Reject the null hypothesis
Combustion Area	Normal	Equal	ANOVA	0.020	Reject the null hypothesis

## Data Availability

The original contributions presented in this study are included in the article. Further inquiries can be directed to the corresponding author.
